# Risk Prediction Model for Esophageal Cancer Among General Population: A Systematic Review

**DOI:** 10.3389/fpubh.2021.680967

**Published:** 2021-12-01

**Authors:** Ru Chen, Rongshou Zheng, Jiachen Zhou, Minjuan Li, Dantong Shao, Xinqing Li, Shengfeng Wang, Wenqiang Wei

**Affiliations:** ^1^National Cancer Center/National Clinical Research Center for Cancer/Cancer Hospital, Chinese Academy of Medical Sciences and Peking Union Medical College, Beijing, China; ^2^Department of Epidemiology and Biostatistics, School of Public Health, Xi'an Jiaotong University Health Science Center, Xi'an, China; ^3^Department of Epidemiology & Biostatistics, School of Public Health, Peking University Health Science Center, Beijing, China

**Keywords:** methodology, risk of bias, systematic review, esophageal cancer, risk prediction model

## Abstract

**Objective:** The risk prediction model is an effective tool for risk stratification and is expected to play an important role in the early detection and prevention of esophageal cancer. This study sought to summarize the available evidence of esophageal cancer risk predictions models and provide references for their development, validation, and application.

**Methods:** We searched PubMed, EMBASE, and Cochrane Library databases for original articles published in English up to October 22, 2021. Studies that developed or validated a risk prediction model of esophageal cancer and its precancerous lesions were included. Two reviewers independently extracted study characteristics including predictors, model performance and methodology, and assessed risk of bias and applicability with PROBAST (Prediction model Risk Of Bias Assessment Tool).

**Results:** A total of 20 studies including 30 original models were identified. The median area under the receiver operating characteristic curve of risk prediction models was 0.78, ranging from 0.68 to 0.94. Age, smoking, body mass index, sex, upper gastrointestinal symptoms, and family history were the most commonly included predictors. None of the models were assessed as low risk of bias based on PROBST. The major methodological deficiencies were inappropriate date sources, inconsistent definition of predictors and outcomes, and the insufficient number of participants with the outcome.

**Conclusions:** This study systematically reviewed available evidence on risk prediction models for esophageal cancer in general populations. The findings indicate a high risk of bias due to several methodological pitfalls in model development and validation, which limit their application in practice.

## Introduction

Esophageal cancer is one of the most common cancers worldwide, with an estimated 6,04,100 new cases and 5,44,076 cancer deaths in 2020 (1). In China, esophageal cancer is the sixth most common cancer and the fourth most common cause of cancer deaths (2). The two primary histologic subtypes of esophageal cancer are esophageal squamous cell carcinoma (ESCC) and esophageal adenocarcinoma (EAC), and squamous dysplasia (SD) and Barrett's esophagus (BE) are known precursors of esophageal cancer for ESCC and EAC, respectively (3). Several factors have been found to be associated with an increased risk for esophageal cancer, including older age, male, family history, smoking, and alcohol consumption (4).

The prognosis of esophageal cancer is poor since most cancers were diagnosed at a late stage. Endoscopic screening plays a key role in the prevention of esophageal cancer. It can reduce the incidence and mortality through early detection, early diagnosis, and early treatment of cancer and precancerous lesions (5, 6). However, population-wide endoscopic screening is less cost-effective due to low detection rate and low compliance, while opportunistic screening is limited in clinical practice due to the high requirement for equipment, technic, and personnel (7).

The risk prediction model is an effective tool for risk stratification and prediction and has been widely used in screening for lung cancer (8), colorectal cancer (9), and breast cancer (10). It is also expected to be applied to esophageal cancer screening to optimize the screening strategy and provide patients with individualized screening guidance. By predicting the probability of esophageal cancer and precancerous lesions in the general population based on predictive factors, risk prediction models can identify high-risk individuals eligible for endoscopic screening, which could concentrate the subjects needed to be screened, reduce the cost and increase the efficiency and compliance of screening, as well as avoid over-screening (11, 12).

In recent years, several risk prediction models with various risk factors have been developed for esophageal cancer. However, it is not clear whether these models are developed or validated appropriately or whether they are applicable to screening for the general population. Therefore, it is important to evaluate the methodology underpinning model development and validation, and also risk factors and performance to guide decisions regarding the use and choice of models for risk predictions. The aim of this systematic review was to provide an overview on the models developed to predict the risk of esophageal cancer in general population, including methodological characteristics, predictors, and risk of bias.

## Materials and Methods

This systematic review was conducted according to the preferred reporting items for systematic reviews and meta-analyses (PRISMA) (13). A pre-specified protocol was followed when this study was conducted.

### Literature Search

We comprehensively searched the PubMed, EMBASE, and Cochrane Library electronic databases to identify relevant publications on December 31, 2020, and updated the search on October 22, 2021. The search strategy consisted of medical subject headings (MeSH) and text words for risk prediction models of esophageal cancer and its precancerous lesions, with the results restricted to human studies. The main search terms were as follows: [(“cancer” OR “carcinoma” OR “neoplasia” OR “tumor” OR “malignancy”) AND (“esophageal” OR “esophagus” OR “gastrointestinal” OR “digestive” OR “alimentary”) OR “esophageal neoplasms” [MeSH]] AND (“predict” OR “predictive” OR “prediction”) AND “model” AND “risk.” Further studies were sought by manually searching for the relevant articles in public databases.

### Selection of Studies

After the removal of duplicates, studies were screened according to the inclusion and exclusion criteria based on titles and abstracts. If a decision could not be made based on abstracts, full articles were retrieved. Studies were selected if they met with the following criteria: (1) published as an original research article in a peer-reviewed journal; (2) developing or validating a risk prediction model of esophageal cancer and/or its precancerous lesions; (3) considering more than one predictors in the prediction model, including traditional and genetic risk factor, laboratory test, or risk scores that combing multiple risk factors; (4) had the outcome as cases with cancer or precancerous lesions vs. non-cases. Two independent authors (RC and JZ) screened the search results to assess conformity with selection criteria, with disagreement resolved with a third author (SW).

### Data Extraction and Synthesis

The same authors independently performed data extraction and quality assessment using standardized protocols. The general characteristics of studies were extracted, including first author, publication year, country, outcome, study design, study period, sample size, and model-related information (statistical methods, model performance, validation method, and included predictors). In this process, the methods of studies published for each risk model were classified according to the TRIPOD guidelines (14). For studies which included multiple models, for example separate models for different outcomes or age groups, all were included separately.

### Assessment of Risk of Bias

The risk of bias was assessed using PROBAST (Prediction model Risk Of Bias Assessment Tool) (15). The tool contains a total of 20 signaling questions to facilitate structured judgment of risk of bias covering four domains on model development and validation: participants, predictors, outcome, and analysis. Any initial disagreement was resolved through further discussion among the authors.

## Results

**Figure 1:**
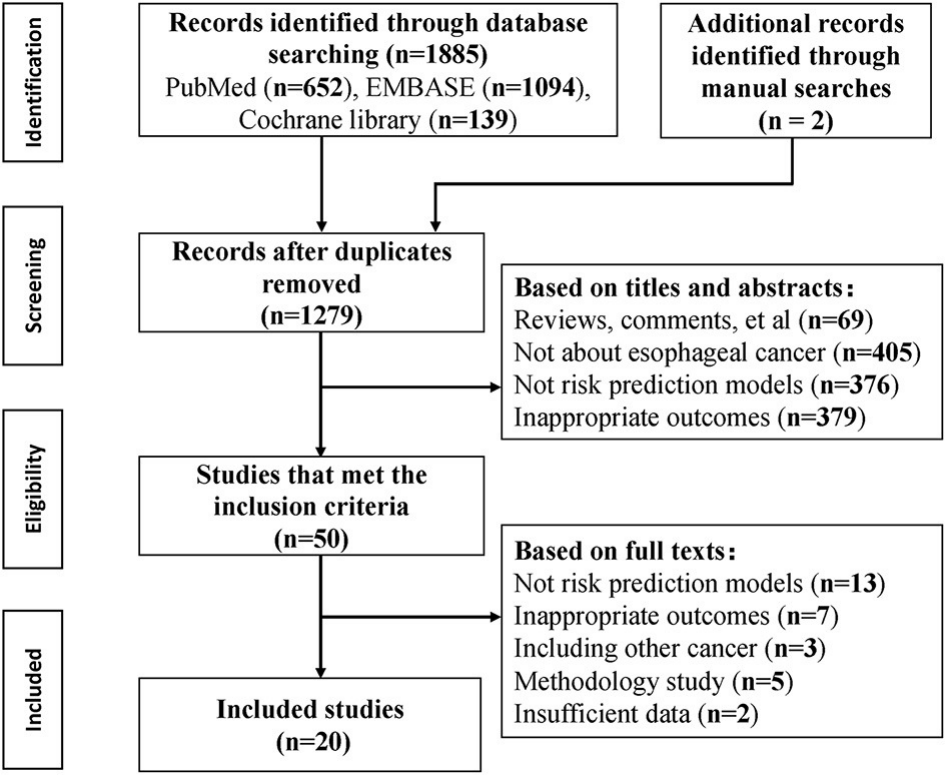
Flowchart of literature search for risk prediction models of esophageal cancer.

The initial search identified 1,885 records, and 20 articles were included according to the inclusion criteria (Figure 1). A total of nine cohorts, 11 case-control studies, and two cross-sectional studies were involved since two studies applied two study designs. Three articles (16–18) reported independent models for different outcomes, three articles reported (19–21) independent models with different predictive factors, one article (22) reported sex-specific models, and one article (11) reported four age-stratified models; thus a total of 30 models were identified on risk prediction for esophageal cancer and precancerous lesions.

### Overview of Risk Prediction Models

Table 1 presents the primary characteristics of the included studies. The publication year ranged from 2008 to 2021. There are 10 models for EAC and BE, 19 models for ESCC and SD, and one model for unspecified esophageal cancer. The sample size of included studies ranged from 355 to 5,55,011, involving 20,776 cases in total. All models on EAC and BE are for people from Western countries, whereas most models on ESCC and SD are for Asians, especially those in East Asian countries including Japan and China. Among the 30 reported models, model discrimination (C statistic) ranged from 0.68 to 0.94, with a median area under the receiver operating characteristic curve (AUC) of 0.78.

**Table 1:** Characteristics of included studies.

**References**	**Country**	**Study design**	**Study type^†^**	**Study period**	**Outcome**	**No. of participants**	**Number of events**	**AUC (95%CI)**
Baldwin-Hunter et al. (23)	USA	Cross-sectional	1b	2015–2017	BE	2,931	57	0.71 (0.64–0.77)
Chang et al. (24)	China	Case-control	2a	NR	ESCC	20,298	9,805	0.71 (0.70–0.72)
Chen et al. (25)	China	Cohort	1b	2007–2012	ESCC	86,447	298	0.81 (0.78–0.83)
Dong et al. (16)	UK	Case-control	2a	NR	BE EAC	7,976	3,288 (BE) 2,511 (EAC)	0.80 (0.78–0.82) (BE) 0.75 (0.73–0.78) (EAC)
Etemadi et al. (17)	Iran	Case-control	2a	2003–2007	ESCC	871 (ESCC)	300 (ESCC)	0.77 (0.74–0.80) (ESCC)
		Cohort	2a	2003–2007	SD	724 (SD)	26 (SD)	0.71 (0.64–0.79) (SD)
Han et al. (26)	China	Cohort	3	2012–2019	ESCC	115,686 (d) 54,750 (v)	186 (d) 120 (v)	0.80 (0.77–0.83) (d) 0.79 (0.75–0.82) (v)
Ireland et al. (27)	Australia	Case-control	1a	2015	BE	355	120	0.82 (0.78–0.87)
Koyanagi et al. (28)	Japan	Case-control	3	2001–2005 (d) 2005–2013 (v)	EC	1,260 (d) 654 (v)	265 (d) 328 (v)	0.94 (9.92–0.95) (d) 0.91 (0.89–0.93) (v)
Kunzmann et al. (18)	UK	Cohort	1b	2006–2010	ESCC EAC	355,034	64 (ESCC) 220 (EAC)	0.71 (0.66–0.78) (ESCC) 0.80 (0.77–0.82) (EAC)
Liu et al. (11)	China	Cohort	2a	2012–2015	SDA MDA	15,073	112 (SDA) 194 (MDA)	0.80 (0.74–0.85) (SAD, < =60 years) 0.68 (0.62–0.74) (SAD, >60 years) 0.77 (MAD, < =60 years) 0.68 (MAD, >60 years)
Liu et al. (29)	China	Cross-sectional Cohort	3	2017–2019	SDA	5,624 (d) 5,767 (v)	87 (d) 34 (v)	0.87 (0.84–0.95) (d) 0.84 (0.79–0.89) (v)
Rubenstein et al. (30)	USA	Cross-sectional	1a	2008–2011	BE	822	70	0.72 (0.66–0.79)
Shen et al. (31)	China	Case-control	2a	2014–2016	ESCC	1,220	244	0.79 (0.75–0.82)
Thrift et al. (12)	Australia	Case-control	2a	2002–2005	EAC	1,944	364	0.76 (0.73–0.79)
Wang et al. (19)	Sweden	Case-control	1b	1994–1997	ESCC	987	167	0.81 (0.77–0.84) (full) 0.79 (0.75–0.82) (simple)
Wang et al. (32)	Norway, UK	Cohort	3	1984–2016 (d) 2006–2010 (v)	ESCC	77,476 (d) 477,535 (v)	53 (d) 105 (v)	0.76 (0.58–0.93) (5 years) (d) 0.70 (0.64–0.75) (5 years) (v)
Xie et al. (20)	Sweden	Case-control	2a	1995–1997	EAC	1,009	189	0.84 (0.81–0.87) (full) 0.82 (0.78–0.85) (simple)
Xie et al. (33)	Norway	Cohort	2a	1995–2015	EAC	62,576	29	0.81 (0.70–0.91) (10 years) 0.88 (0.83–0.93) (15 years)
Yang et al. (22)	China	Case-control	1a	2010–2013	ESCC	3,410	1,418	0.81 (0.79–0.84) (men) 0.88 (0.85–0.90) (women)
Yokoyama et al. (21)	Japan	Case-control	2a	NR	ESCC	868	234	0.86 (HRA-G) 0.84 (HRA-F)

*GC, gastric cancer; ESCC, esophageal squamous cell carcinoma; EC, esophageal cell; EAC, esophageal adenocarcinoma; SD, Squamous Dysplasia; BE, Barrett's esophagus; SDA, severe dysplasia and above (lesions including severe dysplasia and higher-grade lesions); MDA, moderate dysplasia and above (lesions including moderate dysplasia and higher-grade lesions); d, derivation/development; v, validation*.

† *Type of study according to the transparent reporting of a multivariable prediction model for individual prognosis or diagnosis (TRIPOD) guidelines. 1a, development only; 1b, development and validation using resampling; 2a, random split-sample development and validation; 2b, non-random split-sample development and validation; 3, development and validation using separate data; 4, validation study*.

### Predictive Factors

An overview of the predictive factors that were included in the risk prediction models are summarized in Table 2. The number of predictors included in the final models ranged from three to 10, with a median of five predictors. The most common predictors were age, smoking, body mass index (BMI), sex, upper gastrointestinal symptoms, and family history. Besides, genetic variants were included in four studies (16, 21, 24, 28), two of them were ALDH2 genotypes (21, 28), and the other two were single nucleotide polymorphisms (SNPs) or polygenic risk score generated from genome-wide association studies (16, 24).

**Table 2:** Overview of risk factors included in the risk prediction models.

**References**	**Age**	**Sex**	**Smoking**	**Alcohol**	**Family history**	**BMI**	**UGI symptoms^†^**	**Other risk factors**
Baldwin-Hunter et al. (23)		√	√		√^*^		√	
Chang et al. (24)^‡^	√	√	√	√				17 G SNPs and 8 GE SNPs, genetic risk factors interaction with drinking
Chen et al. (25)	√^*^	√^*^	√^*^		√^*^		√^*^	fresh fruit^*^, salted food^*^, relative disease history^*^
Dong et al. (16) (EAC/BE)^‡^	√	√	√			√	√	polygenic risk score, non-steroidal anti-inflammatory drugs
Etemadi et al. (17) (SD/ESCC)^‡^	√		√		√			education, ethnicity, opium use, oral health, marital status, tea temperature, water source
Han et al. (26)	√^*^	√^*^	√^*^	√^*^		√		fresh fruit^*^
Ireland et al. (27)^‡^	√	√		√		√	√	family reflux history, history of hypertension
Koyanagi et al. (28)^‡^	√	√	√	√				ALDH2 genotype
Kunzmann et al. (18) (EAC)	√^*^	√^*^	√^*^			√^*^	√^*^	
Kunzmann et al. (18) (ESCC)	√^*^		√^*^					asthma inhaler use^*^
Liu et al. (11) (SDA, ≤ 60 years)	√^*^					√	√^*^	use of coal or wood as a main source of cooking fuel^*^, rapid ingestion of food
Liu et al. (11) (SDA, >60 years)	√^*^		√^*^		√	√^*^		pesticide exposure^*^, irregular eating habits, high-temperature food intake, eating rapidly, intake of leftover food in summer^*^
Liu et al. (11)(MDA, ≤ 60 years)	√^*^				√^*^		√^*^	use of coal or wood as a main source of cooking fuel^*^, cooking fumes exposure^*^
Liu et al. (11) (MDA, >60 years)	√^*^				√^*^	√^*^		irregular eating habits, high-temperature food intake^*^, eating rapidly, intake of leftover food in summer^*^
Liu et al. (29)	√^*^		√^*^			√^*^	√^*^	
Rubenstein et al. (30)	√^*^		√^*^				√^*^	waist-to-hip ratio
Shen et al. (31)	√^*^	√	√	√^*^				education, intake of hot food^*^, intake of pickled/salted food^*^, intake of fresh fruit^*^
Thrift et al. (12)			√^*^			√^*^	√^*^	education^*^, non-steroidal anti-inflammatory drugs^*^
Wang et al. (19) (full model)	√^*^	√^*^	√^*^	√^*^				education and duration of living with a partner^*^, place of residence during childhood^*^
Wang et al. (19) (simple model)	√	√^*^	√^*^	√^*^				
Wang et al. (32)	√^*^	√	√^*^	√		√		
Xie et al. (20) (full model)			√			√^*^	√^*^	living with a partner for <1 year, previous diagnoses of esophagitis and diaphragmatic hernia and previous surgery for esophagitis, diaphragmatic hernia or severe reflux or gastric or duodenal ulcer
Xie et al. (20) (simple model)			√^*^			√^*^	√^*^	
Xie et al. (33)	√^*^	√	√			√	√^*^	
Yang et al. (22) (men)	√^*^		√^*^	√^*^	√^*^			education^*^, family wealth score^*^, adult height^*^, tooth brushing times^*^, missing and filled teeth^*^, tea temperature^*^
Yang et al. (22) (women)	√^*^				√^*^			education^*^, family wealth score^*^, adult height^*^, tooth brushing times^*^, missing and filled teeth^*^
Yokoyama et al. (21) (HRA-F)			√^*^					alcohol flushing^*^, strong alcohol beverages^*^, green-yellow vegetables^*^, fruit
Yokoyama et al. (21) (HRA-G)			√^*^					ALDH2 genotype^*^, strong alcohol beverages^*^, green-yellow vegetables^*^, fruit^*^

*√Risk factors included in the model*,

* *Significant risk factors*.

† *Including gastroesophageal reflux disease and related symptoms, alarming symptoms of retrosternal pain, back pain or neck pain, epigastric pain and dyspepsia*.

‡ *Significance of risk factors is not reported*.

### Methodology of Risk Prediction Models

The methodological characteristics of the included models are summarized in Table 3. The number of events per variables (EPVs) in the final multivariate analysis was <10 for eight models, and ranged between 10 and 20 for eight models. One third of the models keep continuous variables whereas about half of the models transformed continuous variables to multicategorical variables. Candidate predictors were selected based on multivariable screening in 15 models and clinical experience combined with statistical analysis in 10 models. Eighteen models reported missing values and excluded them from analysis, whereas other studies did not report the issue of missing data. All models reported discrimination with C statistic, of which eight models reported both C statistic and Somers' D statistic. Model calibration was reported in 10 models with Hosmer-Lemeshow test and seven models with calibration plot. Twenty-three models were internally validated using cross validation, bootstrapping, or resampling. Only four models performed independent external validation.

**Table 3:** Methodology of included models.

**Characteristics of analysis**	**Number of studies (%)**
EPV in final model	
<10	8 (26.67)
10–20	8 (26.67)
≥21	14 (46.66)
Handling of continuous variables	
Transforming to multi-categorical variables	16 (53.34)
Keep continuous variables	10 (33.33)
Not report	4 (13.33)
Variable selection	
Multivariable screening	15 (50.00)
Clinical experience and statistical analysis	10 (33.33)
Not report	5 (16.67)
Missing values	
Exclusion from analysis	18 (60.00)
Not report	12 (40.00)
Model performance	
Discrimination	
C statistic/AUC	22 (73.33)
C statistic/ACU and Somers' D statistic	8 (26.67)
Calibration	
Hosmer-Lemeshow test	7 (23.33)
Calibration plot	4 (13.33)
Hosmer-Lemeshow test and calibration plot	3 (10.00)
Not report	16 (53.34)
Validation	
Internal validation	23 (76.66)
External validation	2 (6.67)
Internal and external validation	2 (6.67)
Not report	3 (10.00)
Internal validation (*N* = 25)	
Cross validation	19 (76.00)
Bootstrapping	3 (12.00)
Cross validation and bootstrapping	2 (8.00)
Resampling	1 (4.00)

### Risk of Bias

A summary of the risk of bias analysis is shown in Table 4. None of the models were identified as at low risk of bias. The high risk of bias in participants is mainly due to inappropriate date source, such as cases and non-cases from different populations in case-control studies, which also causes inconsistent definition of predictors and outcomes and makes the domain of predictors and outcome high risk. Major deficiencies in the analysis domain relate to number of participants with the outcome and evaluation of model performance. The relatively recommended prediction model for subjects with high risk of ESCC were Chen's model (25) and Han's model (26), and the model by Kunzmann was recommended for identifying individuals at risk for EAC (18).

**Table 4:** Risk of bias of included studies using PROBAST.

**References**	**Risk of bias**				**Overall**
	**Participants**	**Predictors**	**Outcome**	**Analysis**	
Baldwin-Hunter et al. (23)	Low	Low	Low	High	High
Chang et al. (24)	High	High	High	High	High
Chen et al. (25)	Low	Low	Low	High	High
Dong et al. (16)	High	High	High	High	High
Etemadi et al. (17)	High	High	High	Unclear	High
Han et al. (26)	Low	Low	Low	High	High
Koyanagi et al. (28)	High	Low	Low	High	High
Ireland et al. (27)	High	High	High	High	High
Kunzmann et al. (18)	Low	Low	High	Unclear	High
Liu et al. (11)	Low	Low	Low	High	High
Liu et al. (29)	Low	Low	Low	High	High
Rubenstein et al. (30)	High	Low	Low	High	High
Shen et al. (31)	High	Low	Low	High	High
Thrift et al. (12)	High	High	High	High	High
Wang et al. (19)	Low	High	High	Unclear	High
Wang et al. (32)	Low	Low	Low	High	High
Xie et al. (20)	Low	High	Low	High	High
Xie et al. (33)	Low	Low	Low	High	High
Yang et al. (22)	Low	Low	Low	High	High
Yokoyama et al. (21)	High	High	High	High	High

## Discussion

This systematic review summarizes the available evidence on risk prediction for esophageal cancer in general populations and evaluated the methodology of the included models comprehensively. The findings indicate a high risk of bias in the existing models due in part to limitations in study design and poor reporting and are expected to guide efforts to improve the development, validation, and transparent reporting of risk prediction models for esophageal cancer as well as other related diseases.

Unlike breast cancer (34), colorectal cancer (35, 36), and lung cancer (37), which already have a large number of models with externally validations, the development and validation of risk prediction models of esophageal cancer is still insufficient. Although the included models showed a relatively acceptable performance on discrimination, less than half models evaluated the calibration, and few models have independent external validation. The results of risk of bias assessment indicated that the predictive performance may be overestimated in the derivation dataset and is probably lower than that reported when used in practice. In addition, all EAC/BE models were developed in Western countries, and half ESCC/SD models were developed in China. Due to difference in risk factors among populations, the existing models may not be applicable for people in other high risk areas of esophageal cancer such as Eastern Asia, Southern and Eastern Africa. To ensure the generalizability of models in populations with different characteristics, more models with external validation are needed in further studies (38).

The most common predictors included in the models are well-known risk factors of esophageal cancer, such as age, sex, smoking, and BMI, although there was a variety of predictive factors between different pathological types. For example, ESCC is more related to eating habits including eating rapidly, irregular eating, intake of hot food, intake of fresh fruit, and so on, whereas the more common predictor of EAC is gastroesophageal reflux disease (GERD) (39). However, none of these risk factors are included as predictors by all models. Approximately 80% of the models included age and smoking as predictors, and about half of the models involved sex and BMI. One possible explanation is that these known risk factors did not reach statistical significance in the derivation dataset. A better method to handle these well-established predictors and those with clinical credibility in multivariate modeling is to include them in a prediction model regardless of any statistical significance (40). Some models also incorporate less common predictive factors related to data set features and population characteristics. The predictors only reported by one study include asthma inhaler use (18), waist-to-hip ratio (30), use of coal or wood as the main source of cooking fuel (11), pesticide exposure (24), opium use (17), oral health (12), and alcohol flushing (21). More evidence is needed to verify that their selection is not based on accidental association with esophageal cancer. In addition, there are models that combined genetic risk factors such as genetic polymorphisms or polygenic risk score as predictors to produce better performance. When applied to the general population in both population-based screening and opportunistic screening, the predictors included in the models must be readily obtained in primary care settings. The inclusion of genetic risk predictors may reduce the applicability of the models because they require additional measurement and costs. In summary, the selection of a suitable risk prediction model should comprehensively take the performance, the quality, and the feasibility into consideration.

The results of the risk of bias assessment show that none of the models meet all the requirements of PROBAST (40). The risk of bias in a section of participants, predictors, and outcomes is mainly caused by pitfalls in the study design. In a non-nested case–control study, case patients from a hospital with advanced conditions may be overrepresented, leading to overestimated model performance and reduced model applicability. The methodological limitations in the analysis section are as follows: (1) About 60% of the studies have an EPV lower than 10 and are likely to be overfitting in general (41, 42). For predictors with low-prevalence presented in a model, such as non-steroidal anti-inflammatory drugs (NSAIDs) use (16) and pesticide exposure (11), higher EPV is needed to eliminate bias in regression coefficients and improve predictive accuracy (41). (2) Continuous variables are transformed to multicategorical variables in more than half of the prediction models, the most common of which is categorizing age into groups. However, categorization of continuous predictors might lead to loss of information and weaken the performance of risk prediction models (43). (3) Most of the models either did not report any missing information or exclude missing value in the analysis, which may reduce the available sample size and cause selection bias (44, 45). It should be noted that risk assessment relies on the transparent reporting of studies, and poor reporting could cause the items to be evaluated as high risk or unknown risk. However, to be clear, the clinicians and policy makers, who decide which clinical prediction models should be promoted in evidence-based guidelines or implemented in practice, should refer to reviews like current study at the very beginning. Meanwhile, the high prevalence of models with a high risk of bias emphasizes the need to improve methodological quality of prediction research.

To our knowledge, this is the first review that summarizes the risk prediction models of esophageal cancer in the average-risk population. We used the recently published tool PROBAST to assess the risk of bias and applicability of the included prediction model studies and then evaluated their methodological characteristics and performance. Risk prediction models by Chen (25), Han (26), and Kunzmann (18) were recommended based on the relatively standardized measurement of predictors and outcome, large sample size, complete evaluation, and good result of model performance. The results of this study will provide a reference for the further development, validation, and application of risk prediction models in the target subjects for endoscopic screening.

A limitation of the present study is the methodological shortcomings and inadequate reporting in the included articles. It is not possible to objectively evaluate and directly compare the models based on the poor reporting. Due to the lack of validation studies, we cannot perform meta-analysis of calibration measures for the prediction models. In addition, the variation of predictors and ethnic differences contribute to the poor implementation of the existing models.

## Conclusions

There have been a few risk prediction models developed for esophageal cancer with acceptable predictive ability, although certain methodological limitations, especially the low rate of external validation, should be noted. Further researches are needed to develop a reliable risk prediction model for esophageal cancer based on general population and validate the existing models in an independent dataset.

## Data Availability Statement

The original contributions presented in the study are included in the article/supplementary material, further inquiries can be directed to the corresponding author/s.

## Author Contributions

RC, RZ, SW, and WW designed and supervised the study. RC, JZ, and SW carried out the literature research, extracted data, and wrote the original draft. All authors critically reviewed the manuscript and approved the final draft.

## Funding

This study was supported by the Beijing Natural Science Foundation (7204294), National Natural Science Foundation of China (81903403 and 81974493), and the National Key R&D Program of China (2016YFC0901404 and 2018YFC1311704).

## Conflict of Interest

The authors declare that the research was conducted in the absence of any commercial or financial relationships that could be construed as a potential conflict of interest.

## Publisher's Note

All claims expressed in this article are solely those of the authors and do not necessarily represent those of their affiliated organizations, or those of the publisher, the editors and the reviewers. Any product that may be evaluated in this article, or claim that may be made by its manufacturer, is not guaranteed or endorsed by the publisher.

## References

[1] Sung H Ferlay J Siegel RL Laversanne M Soerjomataram I Jemal A . Global cancer statistics 2020: GLOBOCAN estimates of incidence and mortality worldwide for 36 cancers in 185 countries. CA Cancer J Clin. (2021) 71:209–49. 10.3322/caac.21660 33538338

[2] Zhang S Sun K Zheng R Zeng H Wang S Chen R . Cancer incidence and mortality in China, 2015. J Natl. Cancer Center. (2020) 1:2–11. 10.1016/j.jncc.2020.12.001 PMC11256613 39036787

[3] Lagergren J Smyth E Cunningham D Lagergren P. Oesophageal cancer. Lancet. (2017) 390:2383–96. 10.1016/S0140-6736(17)31462-9 28648400

[4] Abnet CC Arnold M Wei WQ. Epidemiology of esophageal squamous cell carcinoma. Gastroenterology. (2018) 154:360–73. 10.1053/j.gastro.2017.08.023 28823862 PMC5836473

[5] Chen R Liu Y Song G Li B Zhao D Hua Z . Effectiveness of one-time endoscopic screening programme in prevention of upper gastrointestinal cancer in China: a multicentre population-based cohort study. Gut. (2021) 70:251–60. 10.1136/gutjnl-2019-320200 32241902 PMC7815635

[6] Guan CT Song GH Li BY Gong YW Hao CQ Xue LY . Endoscopy screening effect on stage distributions of esophageal cancer: a cluster randomized cohort study in China. Cancer Sci. (2018) 109:1995–2002. 10.1111/cas.13606 29635717 PMC5989864

[7] Kim JA Shah PM. Screening and prevention strategies and endoscopic management of early esophageal cancer. Chin Clin Oncol. (2017) 6:50. 10.21037/cco.2017.09.05 29129090

[8] Ten Haaf K Jeon J Tammemagi MC Han SS Kong CY Plevritis SK . Risk prediction models for selection of lung cancer screening candidates: a retrospective validation study. PLoS Med. (2017) 14:e1002277. 10.1371/journal.pmed.1002277 28376113 PMC5380315

[9] Cooper JA Ryan R Parsons N Stinton C Marshall T Taylor-Phillips S. The use of electronic healthcare records for colorectal cancer screening referral decisions and risk prediction model development. BMC Gastroenterol. (2020) 20:78. 10.1186/s12876-020-01206-1 32213167 PMC7093989

[10] Harkness EF Astley SM Evans DG. Risk-based breast cancer screening strategies in women. Best Pract Res Clin Obstet Gynaecol. (2020) 65:3–17. 10.1016/j.bpobgyn.2019.11.005 31848103

[11] Liu M Liu Z Cai H Guo C Li X Zhang C . A model to identify individuals at high risk for esophageal squamous cell carcinoma and precancerous lesions in regions of high prevalence in China. Clin Gastroenterol Hepatol. (2017) 15:1538–46.e7. 10.1016/j.cgh.2017.03.019 28342951

[12] Thrift AP Kendall BJ Pandeya N Whiteman DC. A model to determine absolute risk for esophageal adenocarcinoma. Clin Gastroenterol Hepatol. (2013) 11:138–44.e2. 10.1016/j.cgh.2012.10.026 23103823

[13] Moher D Liberati A Tetzlaff J Altman DG Group P. Preferred reporting items for systematic reviews and meta-analyses: the PRISMA statement. BMJ. (2009) 339:b2535. 10.1136/bmj.b2535 19622551 PMC2714657

[14] Collins GS Reitsma JB Altman DG Moons KG. Transparent Reporting of a multivariable prediction model for Individual Prognosis Or Diagnosis (TRIPOD). Ann Intern Med. (2015) 162:735–6. 10.7326/L15-5093-2 25984857

[15] Wolff RF Moons KGM Riley RD Whiting PF Westwood M Collins GS . PROBAST: a tool to assess the risk of bias and applicability of prediction model studies. Ann Intern Med. (2019) 170:51–8. 10.7326/M18-1376 30596875

[16] Dong J Buas MF Gharahkhani P Kendall BJ Onstad L Zhao S . Determining Risk of Barrett's esophagus and esophageal adenocarcinoma based on epidemiologic factors and genetic variants. Gastroenterology. (2018) 154:1273–81.e3. 10.1053/j.gastro.2017.12.003 29247777 PMC5880715

[17] Etemadi A Abnet CC Golozar A Malekzadeh R Dawsey SM. Modeling the risk of esophageal squamous cell carcinoma and squamous dysplasia in a high risk area in Iran. Arch Iran Med. (2012) 15:18–21. Available online at: https://pubmed.ncbi.nlm.nih.gov/22208438/22208438 PMC3294378

[18] Kunzmann AT Thrift AP Cardwell CR Lagergren J Xie S Johnston BT . Model for identifying individuals at risk for esophageal adenocarcinoma. Clin Gastroenterol Hepatol. (2018) 16:1229–36.e4. 10.1016/j.cgh.2018.03.014 29559360

[19] Wang QL Lagergren J Xie SH. Prediction of individuals at high absolute risk of esophageal squamous cell carcinoma. Gastrointest Endosc. (2019) 89:726–32.e2. 10.1016/j.gie.2018.10.025 30616974

[20] Xie SH Lagergren J. A model for predicting individuals' absolute risk of esophageal adenocarcinoma: moving toward tailored screening and prevention. Int J Cancer. (2016) 138:2813–9. 10.1002/ijc.29988 26756848

[21] Yokoyama T Yokoyama A Kumagai Y Omori T Kato H Igaki H . Health risk appraisal models for mass screening of esophageal cancer in Japanese men. Cancer Epidemiol Biomarkers Prev. (2008) 17:2846–54. 10.1158/1055-9965.EPI-08-0397 18843030

[22] Yang X Suo C Zhang T Yin X Man J Yuan Z . A nomogram for screening esophageal squamous cell carcinoma based on environmental risk factors in a high-incidence area of China: a population-based case-control study. BMC Cancer. (2021) 21:343. 10.1186/s12885-021-08053-7 33789604 PMC8011400

[23] Baldwin-Hunter BL Knotts RM Leeds SD Rubenstein JH Lightdale CJ Abrams JA. Use of the electronic health record to target patients for non-endoscopic barrett's esophagus screening. Digest Dis Sci. (2019) 64:3463–70. 10.1007/s10620-019-05707-2 31273597 PMC7191846

[24] Chang J Huang Y Wei L Ma B Miao X Li Y . Risk prediction of esophageal squamous-cell carcinoma with common genetic variants and lifestyle factors in Chinese population. Carcinogenesis. (2013) 34:1782–6. 10.1093/carcin/bgt106 23536576

[25] Chen W Li H Ren J Zheng R Shi J Li J . Selection of high-risk individuals for esophageal cancer screening: a prediction model of esophageal squamous cell carcinoma based on a multicenter screening cohort in rural China. Int J Cancer. (2021) 148:329–39. 10.1002/ijc.33208 32663318

[26] Han J Wang L Zhang H Ma S Li Y Wang Z . Development and validation of an esophageal squamous cell carcinoma risk prediction model for rural chinese: multicenter cohort study. Front Oncol. (2021) 11:729471. 10.3389/fonc.2021.729471 34527592 PMC8435773

[27] Ireland CJ Fielder AL Thompson SK Laws TA Watson DI Esterman A. Development of a risk prediction model for Barrett's esophagus in an Australian population. Dis Esophagus. (2017) 30:1–8. 10.1093/dote/dox033 28881896

[28] Koyanagi YN Ito H Oze I Hosono S Tanaka H Abe T . Development of a prediction model and estimation of cumulative risk for upper aerodigestive tract cancer on the basis of the aldehyde dehydrogenase 2 genotype and alcohol consumption in a Japanese population. Eur J Cancer Prev. (2017) 26:38–47. 10.1097/CEJ.0000000000000222 26862830 PMC5142363

[29] Liu Z Guo C He Y Chen Y Ji P Fang Z . A clinical model predicting the risk of esophageal high-grade lesions in opportunistic screening: a multicenter real-world study in China. Gastrointest Endosc. (2020) 91:1253–60.e3. 10.1016/j.gie.2019.12.038 31911077

[30] Rubenstein JH Morgenstern H Appelman H Scheiman J Schoenfeld P McMahon LF Jr . Prediction of Barrett's esophagus among men. Am J Gastroenterol. (2013) 108:353–62. 10.1038/ajg.2012.446 23318485 PMC3903120

[31] Shen Y Xie S Zhao L Song G Shao Y Hao C . Estimating individualized absolute risk for esophageal squamous cell carcinoma: a population-based study in high-risk Areas of China. Front Oncol. (2020) 10:598603. 10.3389/fonc.2020.598603 33489898 PMC7821851

[32] Wang QL Ness-Jensen E Santoni G Xie SH Lagergren J. Development and validation of a risk prediction model for esophageal squamous cell carcinoma using cohort studies. Am J Gastroenterol. (2020) 116:683–91. 10.14309/ajg.0000000000001094 33982937

[33] Xie SH Ness-Jensen E Medefelt N Lagergren J. Assessing the feasibility of targeted screening for esophageal adenocarcinoma based on individual risk assessment in a population-based cohort study in Norway (The HUNT Study). Am J Gastroenterol. (2018) 113:829–35. 10.1038/s41395-018-0069-9 29748563

[34] Anothaisintawee T Teerawattananon Y Wiratkapun C Kasamesup V Thakkinstian A. Risk prediction models of breast cancer: a systematic review of model performances. Breast Cancer Res Treat. (2012) 133:1–10. 10.1007/s10549-011-1853-z 22076477

[35] Peng L Weigl K Boakye D Brenner H. Risk scores for predicting advanced colorectal neoplasia in the average-risk population: a systematic review and meta-analysis. Am J Gastroenterol. (2018) 113:1788–800. 10.1038/s41395-018-0209-2 30315282 PMC6768585

[36] Usher-Smith JA Walter FM Emery JD Win AK Griffin SJ. Risk prediction models for colorectal cancer: a systematic review. Cancer Prev Res. (2016) 9:13–26. 10.1158/1940-6207.CAPR-15-0274 26464100 PMC7610622

[37] Gray EP Teare MD Stevens J Archer R. Risk prediction models for lung cancer: a systematic review. Clin Lung Cancer. (2016) 17:95–106. 10.1016/j.cllc.2015.11.007 26712102

[38] Moons KG Kengne AP Grobbee DE Royston P Vergouwe Y Altman DG . Risk prediction models: II. external validation, model updating, and impact assessment. Heart. (2012) 98:691–8. 10.1136/heartjnl-2011-301247 22397946

[39] Shiota S Singh S Anshasi A El-Serag HB. Prevalence of Barrett's esophagus in asian countries: a systematic review and meta-analysis. Clin Gastroenterol Hepatol. (2015) 13:1907–18. 10.1016/j.cgh.2015.07.050 26260107 PMC4615528

[40] Moons KGM Wolff RF Riley RD Whiting PF Westwood M Collins GS . PROBAST: a tool to assess risk of bias and applicability of prediction model studies: explanation and elaboration. Ann Intern Med. (2019) 170:W1–33. 10.7326/M18-1377 30596876

[41] Ogundimu EO Altman DG Collins GS. Adequate sample size for developing prediction models is not simply related to events per variable. J Clin Epidemiol. (2016) 76:175–82. 10.1016/j.jclinepi.2016.02.031 26964707 PMC5045274

[42] van Smeden M de Groot JA Moons KG Collins GS Altman DG Eijkemans MJ . No rationale for 1 variable per 10 events criterion for binary logistic regression analysis. BMC Med Res Methodol. (2016) 16:163. 10.1186/s12874-016-0267-3 27881078 PMC5122171

[43] Royston P Altman DG Sauerbrei W. Dichotomizing continuous predictors in multiple regression: a bad idea. Stat Med. (2006) 25:127–41. 10.1002/sim.2331 16217841

[44] Vergouwe Y Royston P Moons KG Altman DG. Development and validation of a prediction model with missing predictor data: a practical approach. J Clin Epidemiol. (2010) 63:205–14. 10.1016/j.jclinepi.2009.03.017 19596181

[45] Janssen KJ Donders AR Harrell FE Jr Vergouwe Y Chen Q Grobbee DE . Missing covariate data in medical research: to impute is better than to ignore. J Clin Epidemiol. (2010) 63:721–7. 10.1016/j.jclinepi.2009.12.008 20338724

